# A Quantum Cellular Automata Type Architecture with Quantum Teleportation for Quantum Computing

**DOI:** 10.3390/e21121235

**Published:** 2019-12-17

**Authors:** Dimitrios Ntalaperas, Konstantinos Giannakis, Nikos Konofaos

**Affiliations:** 1 Department of Informatics, Aristotle University of Thessaloniki, Biology Building, Main University Campus, 54124 Thessaloniki, Greece; ntalaperas@csd.auth.gr; 2 Department of Informatics, Ionian University, Tsirigoti Square 7, 49100 Corfu, Greece

**Keywords:** quantum cellular automata, quantum information, cellular automata, quantum gates, quantum circuits

## Abstract

We propose an architecture based on Quantum Cellular Automata which allows the use of only one type of quantum gate per computational step, using nearest neighbor interactions. The model is built in partial steps, each one of them analyzed using nearest neighbor interactions, starting with single-qubit operations and continuing with two-qubit ones. A demonstration of the model is given, by analyzing how the techniques can be used to design a circuit implementing the Quantum Fourier Transform. Since the model uses only one type of quantum gate at each phase of the computation, physical implementation can be easier since at each step only one kind of input pulse needs to be applied to the apparatus.

## 1. Introduction

Feynman [1] was the first to propose that quantum mechanical principles could be harnessed in order to design computers that could, in principle, be more powerful than their classical counterparts, while Margolus [2] demonstrated the existence of quantum-based computational models that could perform reversible computation in the form of Reversible Cellular Automata (RCA). Various quantum computational models, being able to exhibit universal behavior, have also been defined and shown to be equivalent. In particular, Deutsch [3] defined the formulation of the Quantum Turing Machine (QTM), Yao [4] formulated the Quantum Circuit Model and demonstrated its equivalence with the QTM, while Watrous [5] defined one-dimensional Quantum Cellular Automata and showed that there is an efficient way to simulate a subclass of them by a QTM. On the other hand, quantum computation can be described in the prism of multiple angles (e.g., via a logical/algebraic one [6]).

Quantum algorithms, that is, algorithm that rely on quantum computational models, have already been developed and have been shown to outperform their classical analogue. Specifically, Shor [7] designed a quantum algorithm that can factorize composite numbers achieving a superpolynomial speedup, while Grover [8] demonstrated how searching in an unsorted database can be performed by a quantum algorithm with a quadratic speedup. Physical implementation of a quantum computer can pose a challenge, since quantum systems tend to decohere due to unwanted couplings with the environment. Various implementation architectures that try to overcome this problem have however been proposed [9,10].

There is a growing interest on actual implementations of quantum cellular automata, seeking the most appropriate underlying architecture(s) that will facilitate the effective and fault-tolerant functionality of these computational machines. For an in-depth survey on the progress in quantum cellular automata, the interested reader is referred to Reference [11] by Arrighi and [12] by Farrelly. In a recent work, Karafyllidis and Sirakoulis proposed an alternative computational model based on quantum cellular automata [13]. In their approach, the underlying Quantum Cellular Automata (QCA) form a one-dimensional lattice and computation takes place using the idea of quantum walks, that constitutes a proven universal quantum computation model [14]. A similar study was undertaken by Costa et al. in Reference [15], where they showed that two quantum walkers models (the coined and the staggered quantum walkers [14]) can be translated into quantum cellular automata.

One shared characteristics of the vast majority of implementation architectures is that they allow for only neighboring qubits to interact. Due to this limitation, a number of schemes have been studied, where a general quantum algorithm can be converted to an equivalent one that allows only nearest neighbor interactions [16,17]. Typically, these schemes convert a quantum circuit to one consisting of gates acting only on neighboring qubits at the cost of introducing a number of additional gates to the original circuit.

In this paper, an architecture that enforces only nearest neighbor interactions and the application of only one type of a quantum gate per computational step is introduced. This new architecture is based on Quantum Cellular Automata (QCA). The goals of the new architecture are—(a) To convert a generic quantum circuit to an equivalent one having only nearest neighbor interactions and (b) to allow only a specific quantum gate to act on all the qubits for the duration of one computational step. This latter limitation is introduced since in many implementations it is difficult to localize signals corresponding to two different quantum gates. In the architecture presented, a single signal is applied in each step and the same order of error is introduced to all qubit states (since only one type of gate is executed in each step, the error in each qubit state is the same). The only allowed non local interactions are those of quantum teleportation which have been shown to have a nearly zero error rate in various implementation schemes [18].

The present paper is structured as follows: First, an overview of a QCA is given along with an overview of a specific type of QCA proposed by Karafyllidis [19], which operate by applying a single two-qubit quantum gate over the whole quantum register in each step. Afterwards, an overview of nearest neighbor architectures is presented and it is demonstrated how quantum gates that involve qubits over an arbitrary distance, can be performed by using only local interactions and quantum teleportation in a 2D Grid model architecture which is based in ideas introduced by Rosenbaum [20]. Then, we introduce our proposal where the two techniques are combined to this new architecture that retains the advantages of both methods. Similar to the models of Karafyllidis and Rosenbaum, the architecture presented in this work is a high-tier architecture, the benefits of which stem from the way the computations are performed; it can, in theory, be applied to any scheme that allows local qubit interactions and quantum state teleportations. For this new architecture, the execution of an arbitrary quantum gate is demonstrated and the amount of extra operations required per gate is calculated. Finally, an example of developing a quantum circuit implementing the Quantum Fourier Transform (QFT) is given. This demonstrates how a generic quantum computation can be performed in our model.

## 2. Theory

In this Section, the brief description of a quantum cellular automaton (QCA) is provided along with its corresponding operations and circuit models, as proposed by Karafyllidis in Reference [19].

### 2.1. Quantum Cellular Automata

Arrighi et al. [21] gave the axiomatic definition of a QCA by defining QCA as a unitary operator *G* over a quantum labeled graph (QLG). Briefly, a QLG is defined as a tuple

$$\begin{array}{c} \Gamma =(V,E,H), \end{array}$$

with *V* being the nodes, *E* is a subset of V×V that denotes the set of edges and *H* a set of Hilbert spaces being the labels. $H^{x}$ denotes the Hilbert space of node *x* with $\Sigma^{x}$ its possibly infinite countable canonical basis, also known as the alphabet of node *x* [21]. Using this definition, a QCA is defined as a unitary operator over QLG Γ with the following restrictions:

- *V* forming a grid ($V=Z^{n}$)
- *E* connecting nodes *x* to x+z, with $x\in Z^{n}$ and $z\in {\left\{0,1\right\}}^{n}$
- *H* being of finite dimension

Various QCA operational descriptions (e.g., Perez et al. [22]) can be shown to be equivalent to the axiomatic description proposed by Arrighi et al. Descriptions that model the unitary operations as quantum gates constitute can be convenient. Such methods result in quantum circuit structures for QCA.

One such a quantum circuit structure was proposed by Karafyllidis [19]. In this structure, there are two-qubits per cell, the s-qubit (state qubit) and the c-qubit (controlled qubit). At each computational step *t* the state of the jth cell can be written as $|s_{j}^{t}c_{j}^{t}\rangle$. Since there are four basis cell for each cell, the general state of each cell during evolution is:

$$\begin{array}{c} |s_{j}^{t}c_{j}^{t}\rangle =c_{0,j}^{t}|00\rangle +c_{1,j}^{t}|01\rangle +c_{2,j}^{t}|10\rangle +c_{3,j}^{t}|11\rangle , \end{array}$$

with $c_{i,j}^{t}$ being complex numbers. The state of the QCA consists of the tensor product of all the cell states and can be written as

$$\begin{array}{c} |S\rangle ={⊗}_{i=1,n}|s_{i}^{t}c_{i}^{t}\rangle . \end{array}$$

In each step, a controlled operation between a control and a target qubit of adjacent cells takes place, followed by a unitary transformation of the two qubits of each cell. State $S^{t+1}$ is obtained from state $S^{t}$ by applying operator *R* via:

$$\begin{array}{c} |S^{t+1}\rangle =R|S^{t}\rangle , \end{array}$$

with the operator *R* given by:

$$\begin{array}{c} R=R_{e}R_{I}, \end{array}$$

where $R_{e}$ describes the evaluation phase and $R_{I}$ the interaction phase, both given respectively by:

$$\begin{array}{cc} R_{e} & =\;\cdots ⊗U⊗U⊗\cdots \\ R_{I} & =\;\cdots ⊗CN⊗CN⊗\cdots , \end{array}$$

where *U* denotes an arbitrary unitary operation and CN a Controlled-NOT operation.

**Figure 1:**
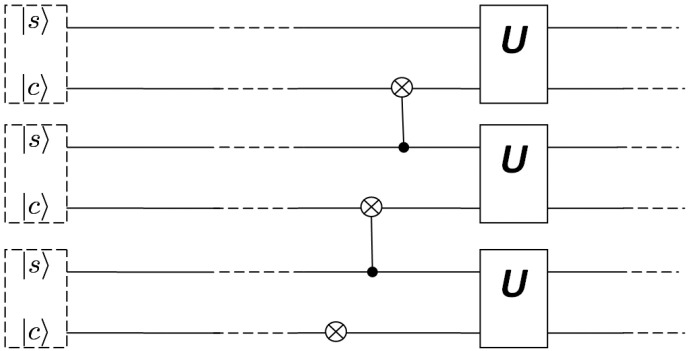
Quantum Cellular Automata (QCA) architecture, as proposed in Reference [7].

The quantum circuit depicted in Figure 1 performs this operation. This architecture exhibits a periodic structure in the output probability patterns.

### 2.2. Single Gate Operations

**Figure 2:**
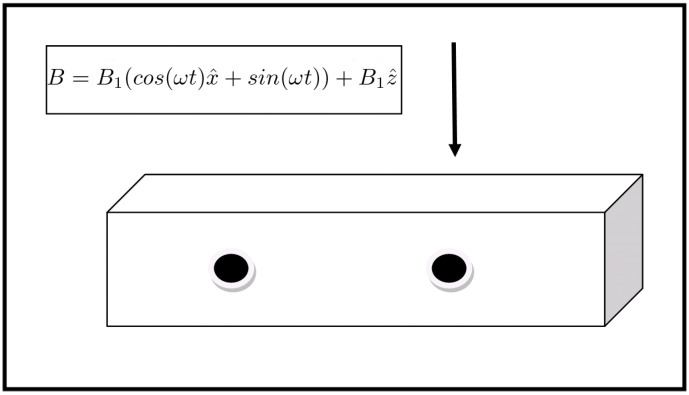
Archetypal spin qubit and qubit operation based on Rabi oscillation.

In a typical scenario, qubits are manipulated by applying a set of external signals to a set of two-level quantum systems that constitute the quantum register. As a typical example, consider the archetypal Rabi oscillation qubit operations depicted in Figure 2. Two spin 1/2 systems $q_{1}$ and $q_{2}$ constitute a quantum register of size two and a magnetic pulse with strength equal to

$$\begin{array}{c} B=B_{1}\left(cos\left(\omega t\right)\hat{x}+sin\left(\omega t\right)\right)+B_{1}\hat{z} \end{array}$$

is applied to qubit $q_{2}$. Let $\omega_{0}=\gamma B_{0}$ and $\omega_{1}=\gamma B_{1}$, with γ being the gyromagnetic ratio. Then, if we choose $\omega =\omega_{0}$ and apply the pulse for a time period of $t=\pi /\omega_{1}$ the equivalent quantum gate of a bit flip is applied to qubit $q_{2}$.

It is the case, however, that the above pulse can, also, alter the state of qubit q1. This is possible, unless: (a) the pulse is extremely localized as to have negligible overlap with q1 or (b) the resonance frequency of the first qubit is significantly different from that of the second or (c) the separation distance of the two qubits is big enough, thus, the field will couple to the spin of the first qubit. Depending on the implementation architecture, the above conditions may be difficult to control. This is attributed to the fact that localized pulses in momentum space may have a spatial extend that overlaps with the wavefunction of an adjacent qubit or to the fact that having different resonance frequencies may require (depending on the system) complex manipulation of the pulse’s parameters. Another contributing factor for this struggle is the use of physical systems that have a different eigenstate spectrum to represent qubits. Increasing the separation distance, on the other hand, may render the interaction between adjacent qubits impossible, thus prohibiting two-qubit operations which are required for universal quantum computing.

All the above conditions may be alleviated if the requirement of performing only one quantum gate to the whole register is imposed in a manner similar to that of Karafyllidis model [19]. The way this is achieved, that is, by converting an arbitrary quantum circuit to an equivalent satisfying this condition (up to some extra teleportation operations) is presented in Section 3.

### 2.3. Nearest Neighbor Circuits

Most implementation techniques allow for only local interactions between qubits, therefore prompting the development of various techniques for converting a generic quantum circuit to one consisting only of gates that act upon neighboring qubits. These techniques typically make use of the SWAP gate and result in a significant increase of the number of quantum gates of the circuit. Saeedi et al. [17] have developed sophisticated algorithms which can reduce the cost of this conversion compared to the naive approaches to a factor up to 82%, with the average improvement being around 50%. On the other hand, Rosenbaum [20] has demonstrated a set of techniques for applying a series of reallocations of qubits in a 2-dimensional grid by using quantum teleportation, so that qubits that are to interact over a distance of more than one qubit apart are transported to adjacent positions.

Allowing for teleportation in two dimensions, may further reduce the cost of adding extra gates for achieving nearest neighbor architecture, since the reordering can be done in fewer operations. Quantum teleportation has been studied and shown to be feasible in various models. D’Ariano et al. [23] have demonstrated the existence of QCA that achieves approximate phase-covariant cloning of qubits while Pfaff et al. [18] have demonstrated the teleportation of arbitrary quantum states between diamond spin qubits over long separations. Of particular interest to QCA is the work of Brennen and Williams [24] who have defined optimal pulse sequences for distributing entanglement over a QCA in a 1-dimensional Ising spin chain.

In the following paragraphs, we give a general description of how a general quantum gate between qubits positioned at arbitrary positions can be performed. Since any quantum gate can be decomposed to a set of gates having at maximum two qubits for input, only interactions between two qubits are considered in this work.

**Figure 3:**
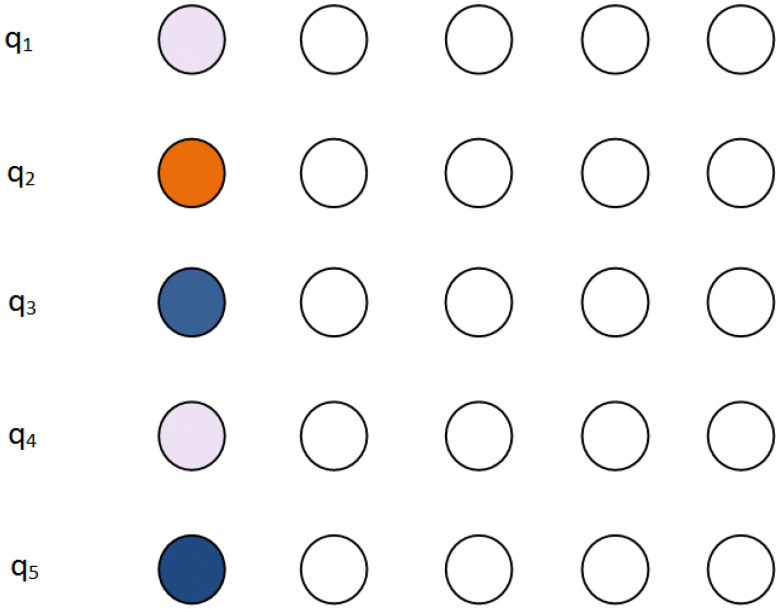
Nearest Neighbor interactions in a two dimensional grid. Initial configuration. Qubits of the same color are to interact with each other while qubits having a unique color will be acted upon by a single-qubit gate.

Figure 3 depicts the initial configuration of a qubit register. The register resides at the leftmost part of the two dimensional grid. Same colors indicate that the two qubits will be the input to the same quantum gate.

In the particular example, qubit 1 is to interact with qubit 4, qubit 3 with qubit 5 and qubit 2 will be operated by a single-qubit operation. Only teleportation in horizontal and vertical directions of the grid should be allowed.

The listing in Algorithm 1 shows the general algorithm for relocating the qubits on the grid. A global position variable holds the current depth to which a qubit will be teleported. For each qubit, it is examined whether it partakes in a quantum gate operation. Two cases are distinguished:

- The qubit partakes in a single-qubit operation. In this case, the qubit is teleported horizontally to position. Position is decreased by one and the qubit is marked as teleported. The case where the qubit partakes in no operation at all is handled the same way.
- The qubit partakes in a two-qubit operation. In that case the qubit is not marked as teleported and it is teleported horizontally to position. The qubit it interacts with is then teleported to position-1. Position is decreased by two and the two qubits are marked as teleported.

**Algorithm 1:** Algorithm for grid rearrangement

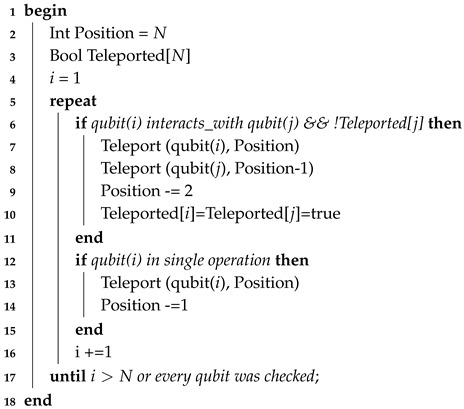



Marking in the above procedure is done classically by marking the index *i* that the qubit has in the quantum register by using classical software.

**Figure 4:**
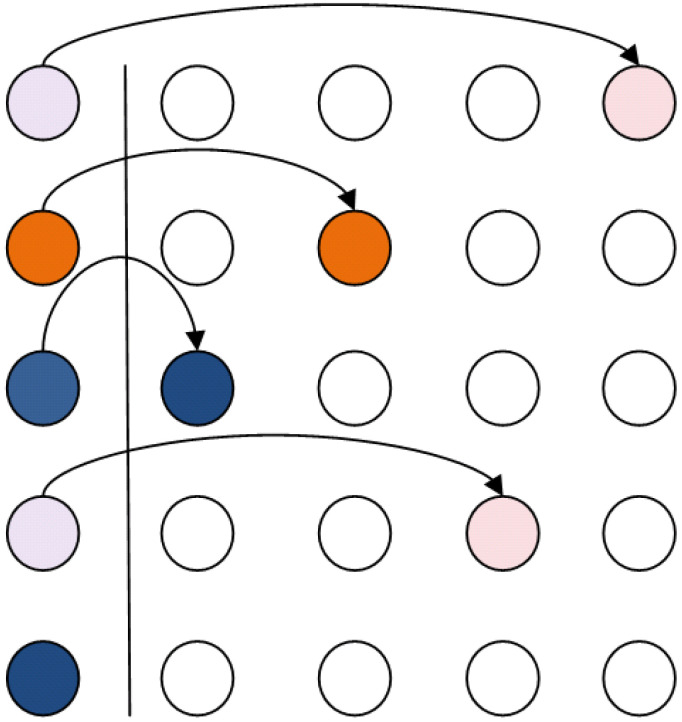
Applying the algorithm for grid rearrangement in an example configuration. Qubits of the same color will partake in a single two-qubit operation. The leftmost column is the initial configuration, after the transposition the qubits are teleported to the right in the position indicated by the corresponding color.

Figure 4 shows a snapshot example of the algorithm when run in the configuration depicted in Figure 3. It is clear that if all the qubits are teleported vertically down to the bottom row, only local interactions are needed in order to execute the computational step required at the starting configuration.

## 3. Arbitrary Quantum Operations

The two models of Karafyllidis and Nearest Neighbor presented before are now combined in order to develop a new model for quantum gate interactions. The goal of the model is twofold:

- Each operation is to be performed only by neighboring qubits.
- In each step of the computation, a single two-qubit gate is to be applied to the whole register holding the current state of the system.

Quantum teleportation is not considered in the set of the above operations, since, as already mentioned, there are various architectures that allow for nearly error-free quantum state transportation. For example, Avalle and Serafini [25] have introduced a class of completely positive maps on a qubit array which generalizes the notion of a QCA allowing it to capture all possible stochastic maps on classical probability distributions. Their work allows for the modelling and computation of error due to dephasing or amplitude damping over a general quantum channel. For entangled states that may typically result from two-qubit quantum operations as the ones supported by our architecture, the situation may be more complex, especially when only partial teleportation (where only a subset of the states is to be teleported) is considered. In this case, Prakash and Verma [26] have demonstrated that the fidelity of the teleportation depends on the information of the input state. Bandyopathyay and Sanders [27] have established lower bounds on the fidelity of the teleportation equal to the fully entangled fraction (FEF) of the Werner state where the FEF is defined as:

$$\begin{array}{c} F\left(\rho \right)=max_{u}\langle \psi^{+}|U^{†}⊗I\rho U⊗I|\psi^{+}\rangle , \end{array}$$

with *U* a unitary state and $\psi^{+}$ the maximally entangled state and the Werner state of a d×d dimensional system being defined as the state satisfying

$$\begin{array}{c} \rho_{AB}=\left(U_{A}⊗U_{B}\right)\rho \left(U_{A}^{†}⊗U_{B}^{†}\right) \end{array}$$

for all unitary operators acting on *d*-dimensional Hilbert space. On the other hand, Yu et al. [28] have demonstrated that when a non-maximally entangled state is used as a quantum channel, then a generic mixed state can be teleported with probability equal to $1-{\left(1-C^{2}\right)}^{1/2}$, where *C* is the concurrence of the quantum channel where the concurrence provides a measure of the channels entanglement [29].

As previously stated, only one-qubit and controlled two-qubit operations are considered. Indeed, such sets of quantum gates (e.g., the Hadamard gate, the π/8 gate and the Controlled-NOT gate) that are suitable for universal quantum operations already exist [30].

Since the mechanism for each case (single-qubit operations and controlled operations) is different, it is preferred to, also, treat each case separately.

### 3.1. Single-Qubit Operations

**Figure 5:**
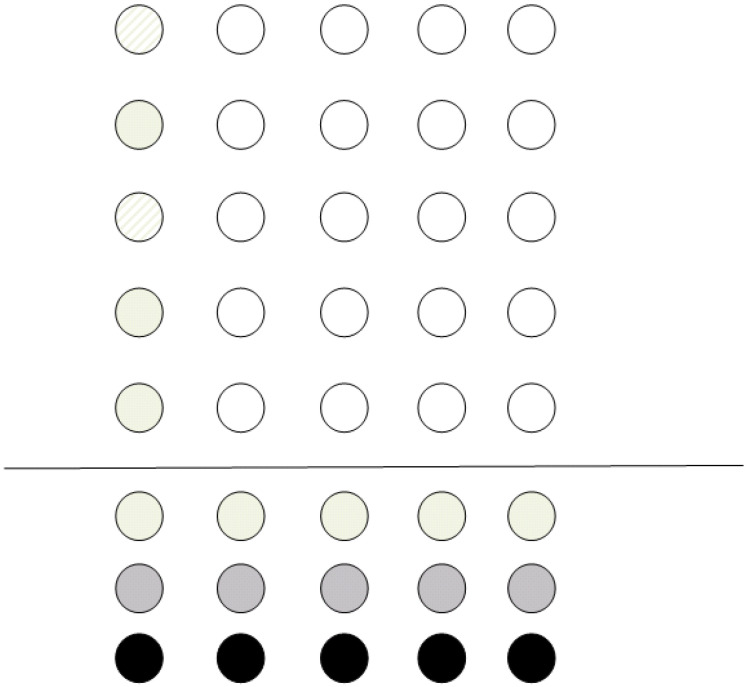
Initial configuration of the computational grid. The horizontal line separates the qubits partaking to the main computation from the ancilla qubits.

Suppose a single-qubit operation is to be performed on some of the qubits. The nearest neighborhood condition is then automatically fulfilled. However, if the operation is applied to the whole register, all the qubits will be altered (even those not partaking to the computation). In order to circumvent this, all single-qubit operations are converted to controlled operations. The goal is to configure the register so as the qubits needed for the computation to be controlled by ancilla qubits (which are initially in prepared states |1〉 and |0〉). Figure 5 shows an example of the configuration of a grid; qubits with dashed lines are those that need to be transformed according to some unitary operation *U*. Grey qubits are ancila qubits prepared in state |0〉, while qubits in black color are prepared in state |1〉.

**Figure 6:**
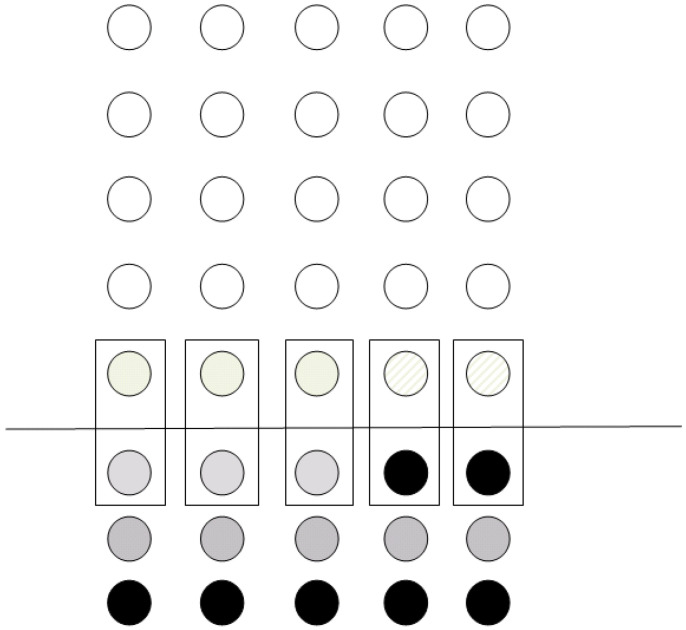
Single-qubit operation–Grid configuration.

In accordance with the procedure described in Section 2.1, the qubits will be teleported to their corresponding positions at the bottom line of the grid. In the next step, the ancila qubits are teleported according to whether the operation is to be performed or not. After all the previous teleportation procedures, the final state of the grid is depicted Figure 6. After the state is prepared, the controlled-*U* operation can be applied simultaneously to the whole register consisting of the data and the teleported ancila data. Thus, by applying a signal operation across the whole register, the equivalent operation of localizing the operation has been achieved at the cost of 3N extra qubits and 2N teleportation operations.

### 3.2. Two-Qubit Quantum Operations

**Figure 7:**
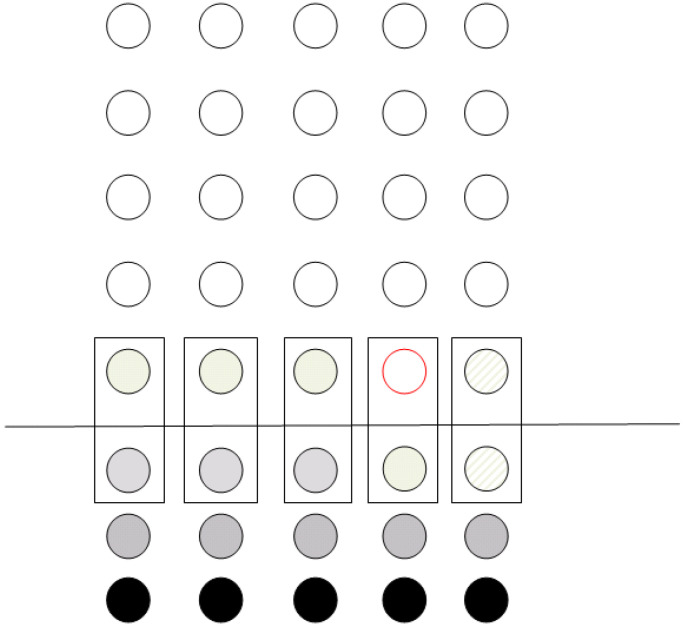
Two-qubit operation–Grid configuration.

For two-qubit operations, the algorithm presented in Section 3 is slightly modified. The second qubit (i.e., the one used as a control) is teleported to the same column as the first qubit. Particularly, it is teleported at the position where the corresponding ancila qubit of the column resides (Figure 7). The controlled operations are performed in a way similar to the one described in Section 3.1, where the qubits not partaking in the operations are used as input to a controlled operation with a control qubit set to state |0〉 via teleportation. The control qubit can then be teleported to the “vacant” spot (circle in red outline in Figure 7) by using two teleportation operations, so that the data register may once again reside in a single line of the grid. The number of operations is the same as those in Section 3.1 plus two extra steps for the final teleportation.

## 4. General Algorithm Flowchart

**Figure 8:**
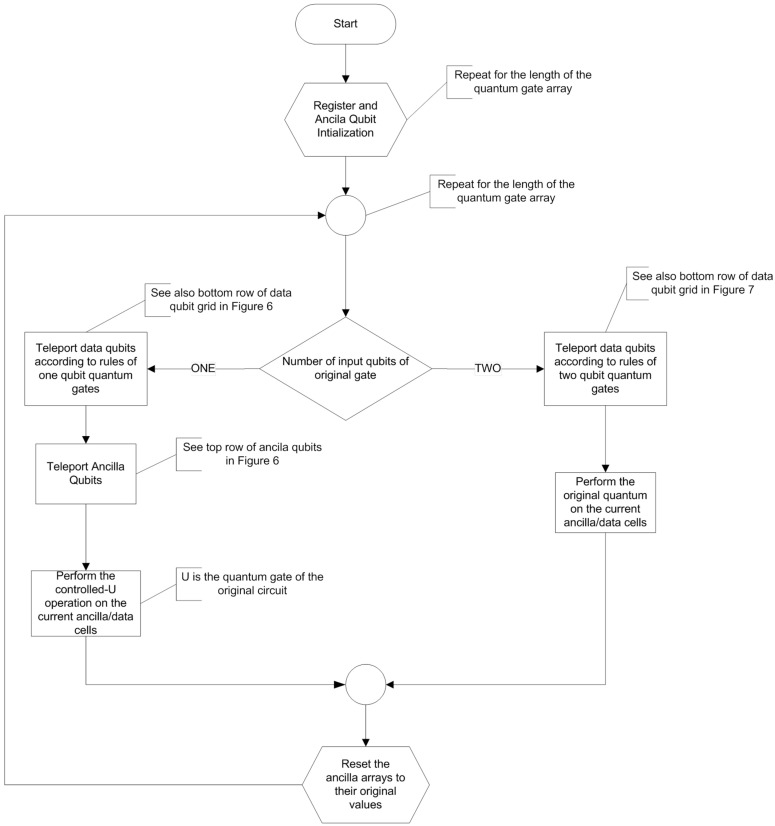
Flowchart of the generic algorithm. Main loop exit and algorithm termination are omitted.

In order to better visualize the execution, the flowchart of the general algorithm is presented in this section, summarizing all the functionalities of the algorithm. The flowchart is depicted in Figure 8. The algorithm loops for *N* iterations, where *N* is the size of the extended original quantum circuit. Extended circuit in this context means that quantum gates are performed one at a time, except for the case that the same gate is applied to different places in the quantum register. In the latter case, these gates are grouped in the same step in the extended circuit.

The algorithm converts the computational step of each gate to a set of equivalent gates consistent with the rules presented so far.

- If the quantum gate is a single-qubit gate, the transformation presented in Section 3.1 (Figure 5) is applied
- If the quantum gate is a two-qubit gate, the transformation presented in Section 3.2 (Figure 6) is applied

This process continues for *N* steps and the final measurement is performed to the working register. It is essential to restore any ancila registers to their initial status, since they will be used again each time the working register is rotated back to the same position.

## 5. Application–Quantum Fourier Transform

**Figure 9:**
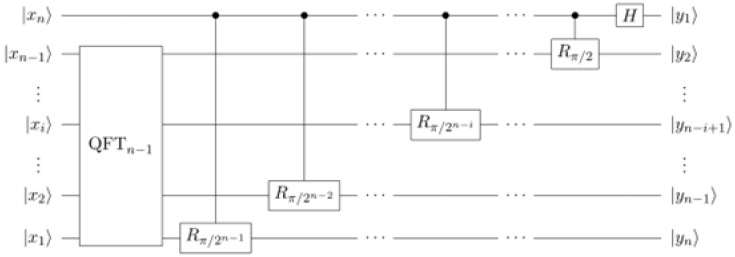
The Quantum Fourier Transform (QFT) circuit.

Quantum Fourier Transform (QFT) resides at the core of Shor’s algorithm. The circuit implementing the Quantum Fourier Transform is demonstrated recursively in Figure 9. In each step, qubit *n* is used as a control for controlled phase operations on each of the other qubits. A single Hadamard transformation is applied to qubit *n* at the end.

To demonstrate how the proposed architecture works in a generic scenario, we will show the details of how a sequence of controlled-phase operations and a Hadamard gate are performed. Actually, this can be extended to the complete QFT circuit, since QFT^n^ consists of n-1 controlled-phase operations plus a Hadamard operation.

**Figure 10:**
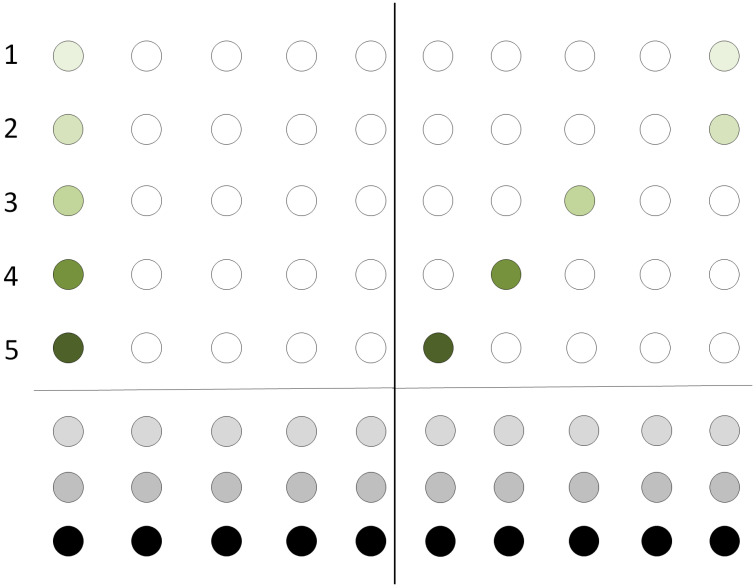
Controlled $R_{\pi /2}$. From initial configuration (left side) to the first horizontal teleportation. Qubits 1 and 2 will interact so they are teleported to the same column.

To begin with, we consider the state of the register where each qubit is in an unknown superposition which depends on the previous computational steps. For the case of a five-qubit register, this is depicted in the left side of Figure 10. Each qubit is represented with a different shade of green color. This shade is used to mark the position of the qubit throughout the computation and is not representative of the qubit’s state.

At first, the controlled $R_{\pi /2}$ gate is to be performed between qubits at row positions 1 and 2. In accordance with the algorithm for performing a two-qubit gate, the qubits are first teleported horizontally as displayed in the right side of Figure 10.

**Figure 11:**
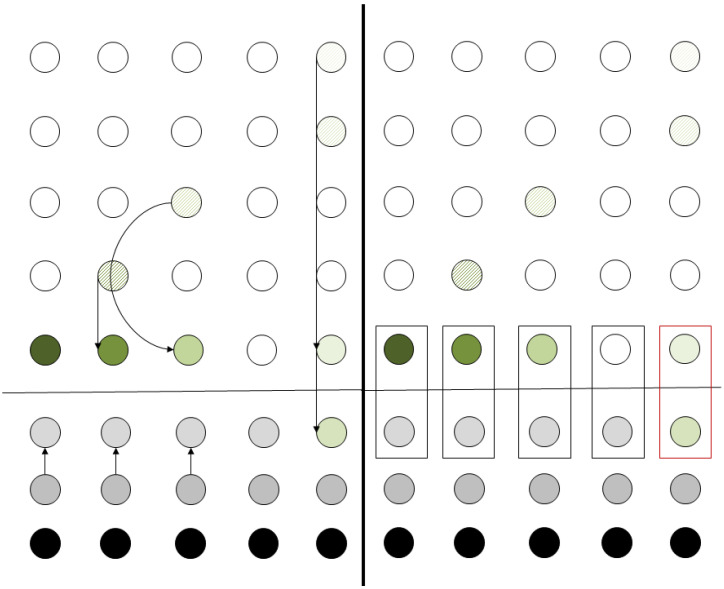
Controlled $R_{\pi /2}$. Vertical teleportation and the application of the two-qubit quantum operation.

The process continues by vertical teleportation and the grid reaches the configuration depicted on the left side of Figure 11. In the same instance, we demonstrate the teleportation of qubits in state |0〉 to the relevant positions in the ancila qubits row. The ancila qubit in column 4 is not used and, thus, the corresponding teleportation is not performed.

After the vertical teleportation, the grid has reached a configuration that allow the actual computation to take place. This is shown on the right side of Figure 11. Each box represents a controlled $R_{\pi /2}$ operation. The first four operations leave the computational qubits unaffected, since the ancila qubit (which is used as a control) is in state |0〉. The last operation (denoted by the box in red outline in the right side of Figure 11) is the actual $R_{\pi /2}$ operation between qubits 1 and 2 that is required by QFT. All these operations are performed in parallel, so that in each step, a single two-qubit operation is performed both to the whole register and the ancilla qubit array. As required by the model to perform the computation, only nearest neighbor operations are applied, whilst a single kind of signal is required, too.

**Figure 12:**
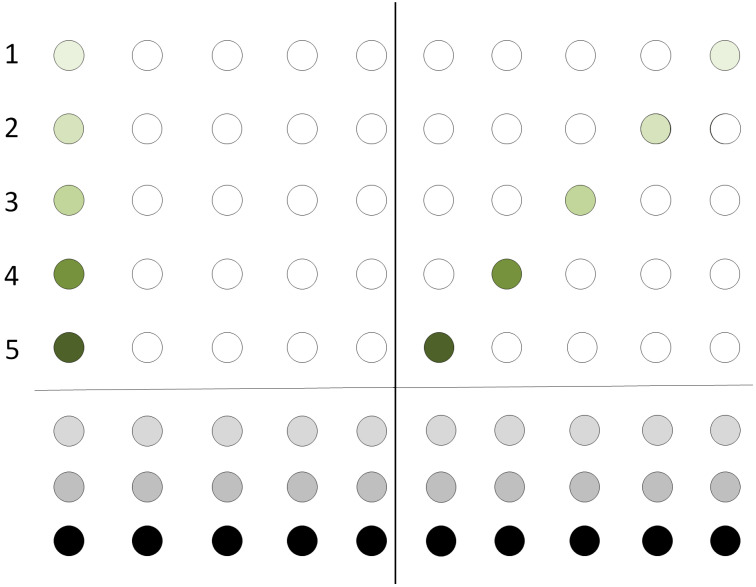
Hadamard Gate. From initial configuration to the first horizontal teleportation.

Then, in order to complete the computation, the Hadamard gate must be performed on the first qubit. At first, the cleanup actions of the previous operation must be performed. This, as described in Section 3.2, involves the teleportation of qubit 2 to the “vacant” position of the register. After the teleportation, the register that now contains the original qubits can be used to initiate the procedure needed for the Hadamard gate. This initial configuration is depicted in the left side of Figure 12. The grid is depicted “rotated”. This rotation is not physical but rather a visual cue to help visualizing the process; the qubits should be considered to occupy the same places in the grid as before.

In the right side of Figure 12, the configuration of the grid after the first horizontal teleportation may be seen. This step is similar as before, the exception being that now all qubits are teleported in their respective columns since no qubit will act as control.

**Figure 13:**
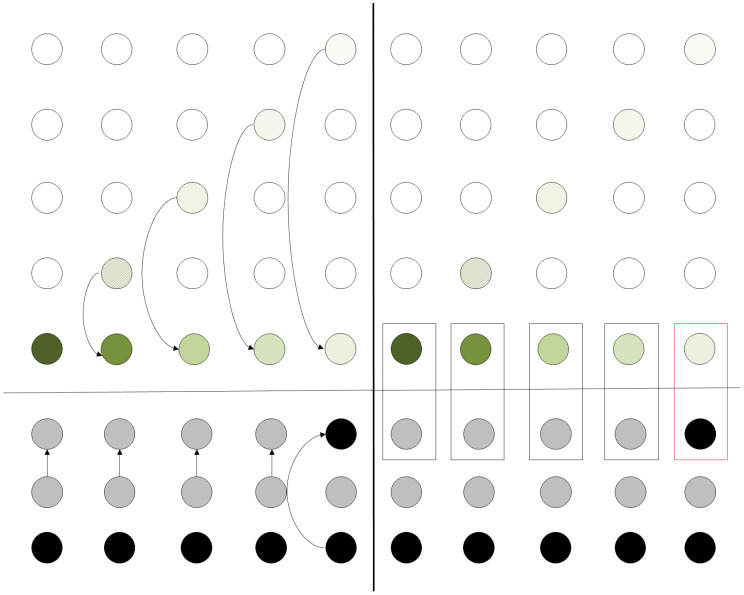
Hadamard gate. Vertical teleportation and applying the gate using control qubits in pre-prepared states.

The algorithm proceeds with the vertical teleportation, as can be seen in the left side of Figure 13. In parallel, the needed ancila qubits are teleported. For this operation, the ancila qubits of the first four columns are set to state |0〉 by teleporting qubits already set to state |0〉. This ensures that the state of the corresponding qubits in the working register will remain unaltered. For the 5th column, a qubit in state |1〉 is teleported in the corresponding ancila position. Similarly to the previous gate, a controlled Hadamard is performed in a pairwise fashion to all qubits of the working register and the ancila register. Since only the qubit of the fifth column is affected, this operation is equivalent to a Hadamard gate performed on said qubit.

Therefore, it has been demonstrated that the proposed model is at least capable of performing any computation that can be described by a Quantum Circuit and is, in this sense, Turing complete (since the quantum circuit model has already been shown to be Turing-complete [31,32]).

For computing the total additional space and number of steps that the model introduces compared to a quantum circuit of size *N*, we note the following:

- The grid needs $N^{2}$ working qubits plus 4N qubits for the row of the ancila qubits. An additional 2N qubits holding prepared states of |0〉 and |1〉 are needed if we wish to perform all the ancila teleportation operations in parallel.
- Each computational step needs *N* horizontal teleportation operations plus *N* vertical (the extra two teleportation in the case of two-qubit gates can be ignored). There are, also, at max *N* teleportation operations for the ancila qubits plus at max *N* initialization operations, since we need the qubits holding the prepared states to be returned to their original states after the teleportation operations. Finally, there are *N* controlled operations in each phase of the computations.

The above calculations yield a cost of additional $O(n^{2})$ space and O(n) additional steps. However, as has been demonstrated, the additional steps can be performed in parallel with each operation being executed in three phases. Phase one consists of the initial horizontal teleportation, phase two contains the vertical teleportation and phase three consists of the quantum controlled operations (ancila teleportation operations and re-initialization may be performed during phases one and/or two).

The benefit from all these, however, is that the model permits only nearest neighbor quantum operations which can be performed using localized signals. Furthermore, a single kind of signal is performed during each computational step. It should be noted that, ignoring quantum teleportations (which can be regarded as qubit transfer operations), the depth of the original circuit does not change despite the introduction of extra quantum gates. This is due to the parallel execution of these gates in each step and it is in contrast with various nearest neighbor architectures that require an increase in the quantum circuit’s depth in the general case. Including teleportations, the increase of circuit depth is up to a constant per each step.

### Flowchart of Quantum Fourier Transform Operations

**Figure 14:**
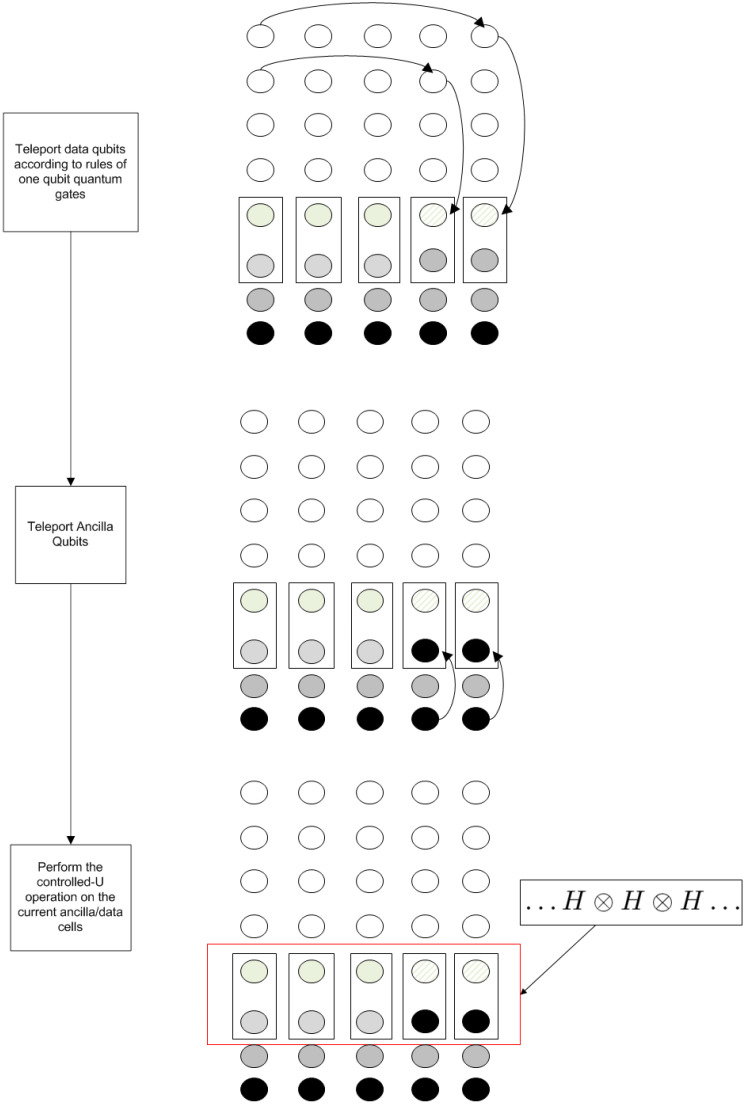
Stages for the execution of the Hadamard gate. It is implied that the operation performed in the last stage is the controlled Hadamard operation.

**Figure 15:**
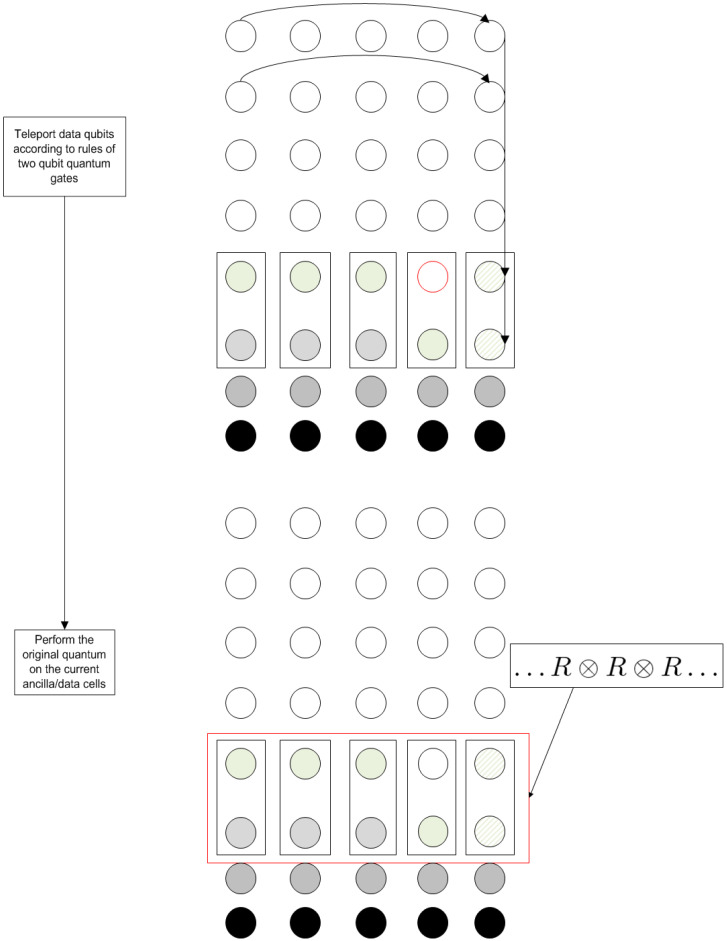
Stages for the execution of the controlled-Phase operation.

The Quantum Fourier Transform consists of a set of Hadamard and controlled-Phase operations. The various stages of the execution of each gate and their correspondence with the new algorithm’s flowchart are depicted in Figure 14 (single-qubit operations) and Figure 15 (two-qubit operations).

Each of the two possible sets of operations of the main loop of the algorithm is mapped into the state of the computational grid with arrows denoting teleportation operations. As can be seen, apart from these teleportations, only one type of quantum gate is applied in the last stage of the procedure to the whole working register.

## 6. Conclusions

In this paper, a novel architecture has been proposed with the aim of combining the techniques of Quantum Cellular Automata and Nearest Neighbor interactions. The proposed architecture benefits from the advantages of both techniques; QCA model offers simplicity in design and the capability of applying a gate array consisted of similar gates, whereas the Nearest Neighbor model offers an easier implementation scheme and a low error rate overhead.

While the proposed architecture requires that some extra computational steps and qubits have to be introduced (of linear and quadratic order respectively), this is compensated by the fact that the underlying operations are performed in a local and controlled manner, which in turn yields the decrease of the error rate of each computational step.

## Abbreviations

The following abbreviations are used in this manuscript:

QCA	Quantum Cellular Automata
QTM	Quantum Circuit Model
QFT	Quantum Fourier Transform
QLG	Quantum Labeled Graph
